# Supplementary material for: Intracellular Staphylococcus aureus Elicits the Production of Host Very Long-Chain Saturated Fatty Acids with Antimicrobial Activity
Source: Metabolites. 2019 Jul 20;9(7):148. doi: 10.3390/metabo9070148 (PMC6680556; doi:10.3390/metabo9070148)
Supplement: Supplementary file 1 [file metabolites-09-00148-s001.pdf]

Supplementary figures

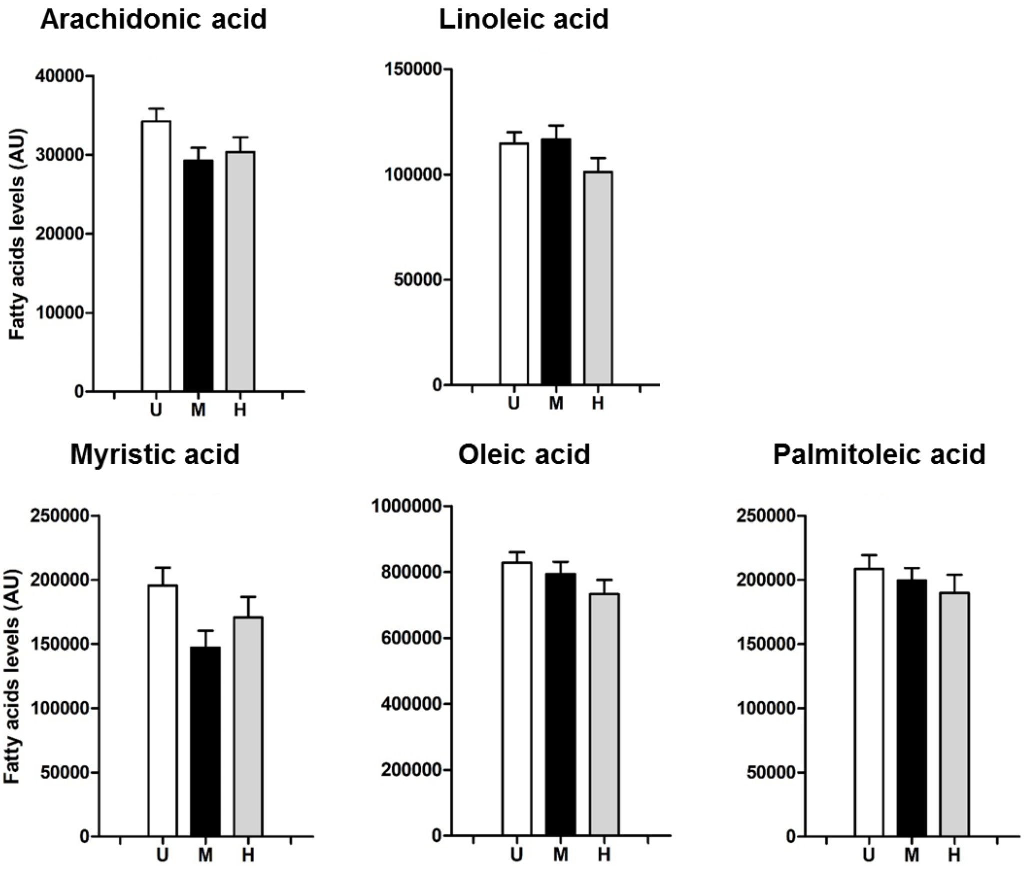

Figure S1. Absolute levels of arachidonic, linoleic, myristic, oleic and palmitoleic acids after intracellular USA300 infection. HeLa cells were infected with USA300 and exposed to heat-killed USA300 strains (MOI 100; 6 hours) and fatty acids levels were detected by GC-MS. Bar graphs show absolute levels of each fatty acid in uninfected cells (white bars), cells infected with USA300 strain (black bars) and cells exposed to heat-killed USA300 strain (grey bars). Data are expressed as means ± standard error (SE) of three independent experiments performed in triplicates. One-way ANOVA and post hoc Tukey´s multiple comparison tests were performed to assess statistically significance across treatments. No significant differences were found between treatments for any of these six fatty acids. U=Uninfected cells; M=MRSA-infected cells; H=cells exposed to heat-killed bacteria.

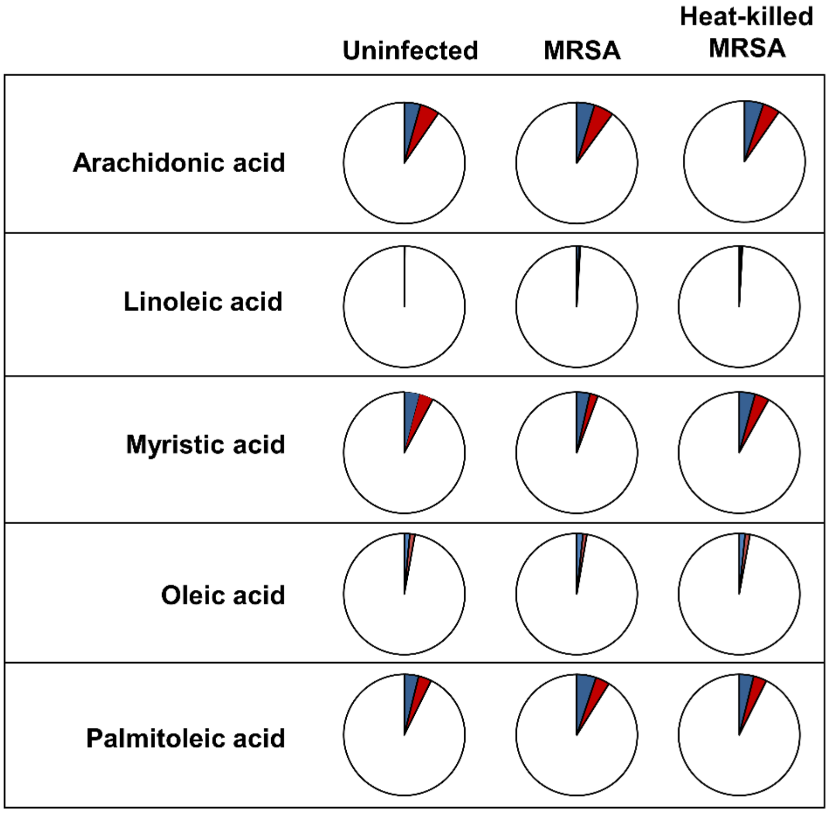

Figure S2. Labelling distribution of arachidonic, linoleic, myristic, oleic and palmitoleic acids after intracellular USA300 infection. HeLa cells were infected with USA300 and exposed to heat-killed USA300 strains (MOI 100; 6 hours) and labelling distribution was detected by GC-MS. Pie charts display the labelling pattern of each fatty acid in uninfected cells (left pie-chart), cells infected with USA300 strain (middle pie-chart) and cells exposed to heat-killed USA300 strain (right pie-chart). Within pie-charts, blue slides represent carbon coming from glucose-labelled, whereas red slides are carbon coming from glutamine-labelled and white slides show carbon from other sources.

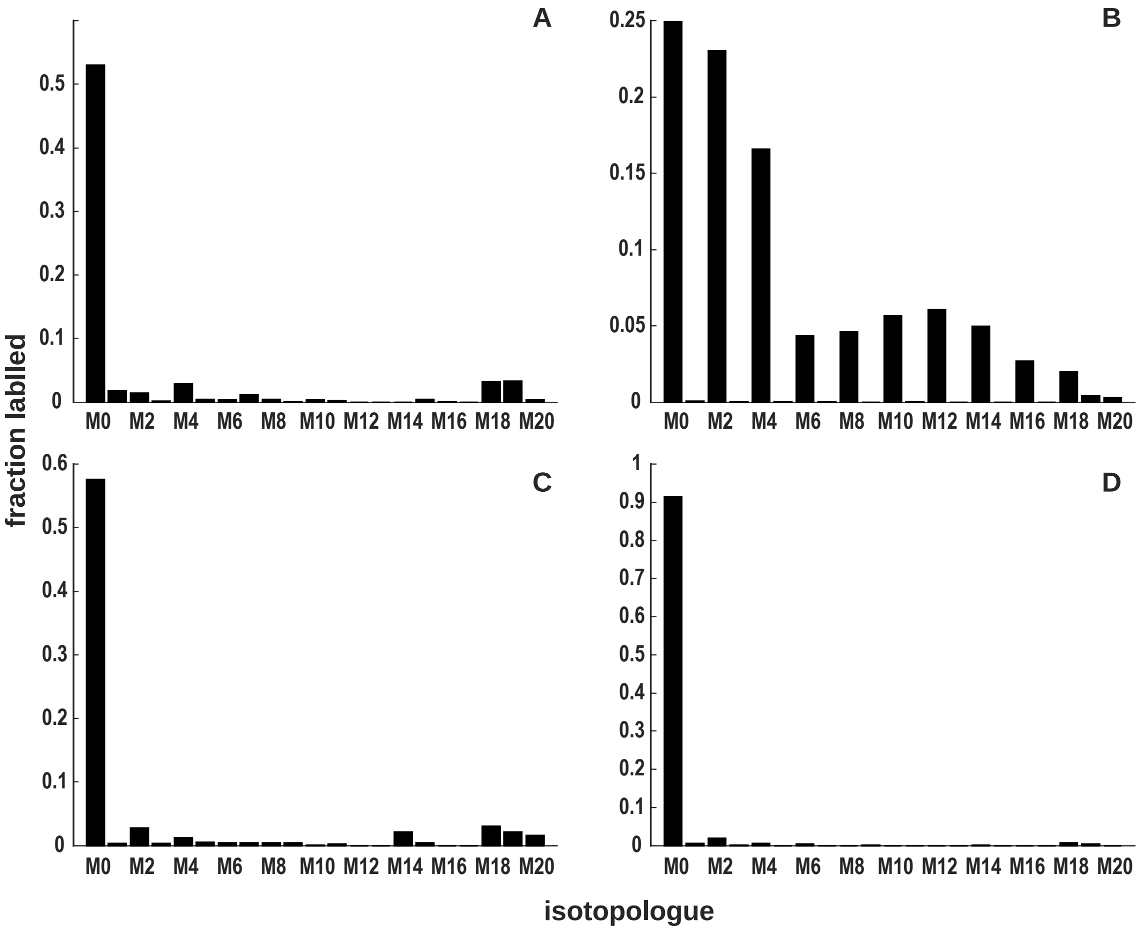

Figure S3: Isotopologue distribution of eicosanoic acid. HeLa cells, uninfected (**A**/**C**) or infected with USA300 (MOI 100; 6 hours, **B**/**D**), were grown in the presence of $^{13}C_6$ glucose (**A**/**B**) or $^{13}C_5$ glutamine (**C**/**D**). Labelling distribution was detected by GC-MS and quantified using an in-house workflow incorporating natural isotope correction. Contaminating signal for *m+13* and *m+17* were removed prior to plotting for clarity. These signals contributed combined relative errors of 4% and 12% for infected and uninfected samples, respectively.
